# Association Between Obesity and Thrombosis During Pregnancy and the Postpartum Period: A Case-Control Study From a Tertiary Care Center in Kerala, India

**DOI:** 10.7759/cureus.75511

**Published:** 2024-12-10

**Authors:** Indhumathi Shanmugam, Nimmi Varghese, Harini Sivamani

**Affiliations:** 1 Obstetrics and Gynecology, University Hospital of North Tees, North Tees and Hartlepool NHS Foundation Trust, Stockton-on-Tees, GBR; 2 Obstetrics and Gynecology, Government Medical College, Thiruvananthapuram, IND; 3 Obstetrics and Gynecology, Stoke Mandeville Hospital, Buckinghamshire Healthcare NHS Trust, Aylesbury, GBR; 4 Urogynecology, Advanced Center for Urogynecology Private Limited, Chennai, IND; 5 Urogynecology, Chennai Urology and Robotics Institute Private Limited, Chennai, IND

**Keywords:** body mass index, cortical venous thrombosis, deep vein thrombosis, obesity, pulmonary thromboembolism

## Abstract

Background

Obesity is postulated to be a high-risk factor for thrombosis along with the inherent hypercoagulability of pregnancy. The Confidential Review of Maternal Deaths (CRMD) found that thrombosis was one of the major causes of maternal deaths in Kerala. This study investigates the major risk factor - obesity and its association with thrombosis in our study setting, along with other risk factors. Obesity, being a modifiable risk factor, can thus be targeted to decrease maternal morbidity and mortality for a better pregnancy outcome. This study sought to examine the association between obesity and thrombosis during pregnancy and the postpartum period in a tertiary care center in Kerala, India, while also identifying other risk factors that may contribute to thrombotic events in this population.

Methodology

A hospital-based case-control study was conducted at Government Medical College, Thiruvananthapuram, Kerala, India, from March 2017 to September 2018. The study population comprised 42 cases diagnosed with thrombotic events during pregnancy and the postpartum period, including deep vein thrombosis (DVT), cortical venous thrombosis (CVT), and pulmonary thromboembolism (PTE). Using consecutive sampling from the hospital's labor register, 84 controls were selected, maintaining a case-to-control ratio of 1:2. Detailed clinical information was collected through direct patient interviews and medical record reviews. Body mass index (BMI) was calculated using pre-pregnancy weight and height measurements. Variables assessed included demographic characteristics, obstetric history, socioeconomic status, blood group, gestational age at thrombosis, type and location of thrombosis, risk factors, comorbidities, clinical manifestations, treatment modalities, and outcomes.

Results

The study demonstrated a significant association between obesity and thrombotic events in pregnancy and postpartum periods. In the case group, 21 patients (50.0%) presented with Class I obesity, and three patients (7.1%) with Class II obesity. The control group included 14 patients (16.7%) with Class I obesity and no patients (0%) with Class II obesity, revealing a significant difference in BMI class distribution between groups (χ^2^=25.979, p<0.001). Among the cases, DVT was the predominant presentation in 29 patients (69.0%), primarily affecting the ileo-femoral region in 26 patients (61.9%). CVT occurred in 12 patients (28.6%). Of the DVT patients, obesity was present in 17 patients (58.6%) compared to 12 non-obese patients (41.4%), showing statistical significance (χ²=14.488, p=0.001). Additional significant risk factors were identified in the study group: period of immobility affected five patients (11.9%), puerperal infection was present in five patients (11.9%), and antiphospholipid antibodies (APLA) positivity was found in five patients (11.9%). All these risk factors showed statistically significant differences compared to controls (p=0.001). Treatment outcomes were favorable, with 40 patients (95.2%) achieving either complete resolution or showing improvement.

Conclusion

Obesity significantly increases pregnancy-associated thrombosis risk, particularly DVT in the ileo-femoral region. Additional factors like immobility, puerperal infection, and APLA positivity contribute to thrombosis development. While treatment outcomes are favorable, early recognition and management are crucial, especially in women with multiple risk factors.

## Introduction

Obesity is a chronic, multifaceted condition characterized by excessive adipose tissue that may adversely affect health. It may elevate the risk of type 2 diabetes and cardiovascular disease, impact bone health and reproductive functions, and heighten the likelihood of some malignancies [1]. Obesity has become a significant global health concern, with the World Health Organization indicating that global obesity rates have almost quadrupled since 1975. In 2022, over 1.9 billion people were classified as overweight, with 650 million of them being obese [1]. The National Family Health Survey-5 (2019-2021) indicates that the overall prevalence of obesity among women of the reproductive age group (15-49 years) in India has gone up from 20.6% to 24%, with Kerala exhibiting one of the highest rates at 38.1% [2]. Research conducted at Indian tertiary care facilities indicates that maternal obesity correlates with a greater chance of negative pregnancy outcomes, including thrombotic problems [3].

The association between obesity and thrombosis involves complex mechanisms. Obesity induces a prothrombotic state characterized by increased inflammation, enhanced oxidative stress, and significant alterations in coagulation factors [4]. These changes include elevated levels of factor VIII, von Willebrand factor, and fibrinogen, alongside decreased fibrinolytic activity. During pregnancy, this risk is further amplified due to the physiological hypercoagulable state [5].

Population-based research has shown that maternal obesity enhances the risk of venous thromboembolism (VTE) throughout pregnancy and the postpartum period by 2.5 to 5.3 times relative to women of normal weight [6]. The pathophysiological mechanisms underlying this association involve multiple pathways, including endothelial dysfunction, increased levels of procoagulant factors, and impaired fibrinolysis [7]. Recent research has revealed that pre-pregnancy overweight and obesity have long-lasting effects, significantly increasing the risk of VTE even years after pregnancy [8].

The management of thrombosis in obese pregnant women presents unique challenges, including compromised diagnostic accuracy due to body habitus affecting imaging quality and inadequate standard prophylactic anticoagulation dosing [9]. A study conducted by Sahu et al. in India revealed that obese pregnant women had markedly elevated risks of complications necessitating specialist care and treatments [10].

The CRMD report indicates that thrombosis is among the primary causes of maternal mortality, contributing to a significant number of preventable deaths in the state of Kerala [11]. Although a consistent association between obesity and antepartum VTE has not been established, postpartum thrombosis demonstrates a higher incidence among individuals with a body mass index (BMI) exceeding 35 [12].

Comprehending this relationship is of paramount importance, as obesity constitutes a modifiable risk factor, and interventions aimed at weight management may effectively mitigate the risk of thrombotic events [13]. This knowledge provides valuable insights that can inform the development of clinical practice guidelines targeted at the local population. This study aims to determine the association between obesity and thrombosis during pregnancy and the postpartum period at a tertiary care center in Kerala, India, while also identifying other critical risk factors that might influence the occurrence of thrombotic events in this patient population.

## Materials and methods

Study design and setting

This hospital-based case-control study was conducted in the Department of Obstetrics and Gynecology at Government Medical College, Thiruvananthapuram, Kerala, India, from March 2017 to September 2018.

Cases

All diagnosed cases of thrombosis (including deep vein thrombosis (DVT), cortical venous thrombosis (CVT), and pulmonary thromboembolism (PTE) during pregnancy and the postpartum period were admitted to the hospital during the study period.

Controls

For each case, two controls were selected from the labor register of the same hospital using consecutive sampling.

Inclusion criteria

All diagnosed cases of thrombosis in pregnancy and puerperium period throughout the research period were included.

Exclusion criteria

Patients who declined to participate in the investigation were excluded.

Sample size estimation

The sample size for this study was calculated using Kelsey et al. formula for an unmatched case-control study [14]. Based on a confidence level of 95% and a power of 80%, the sample size was determined with parameters such as case-to-control ratio (1:2) and odds ratio. The odds ratio of 5.3 was referenced from a previous population-based nested case-control study by Larsen et al., which found that maternal obesity increased the risk of VTE during pregnancy and the postpartum period by over five times compared to normal-weight women [4]. This study reported a 4.7% prevalence of obesity among the control group of pregnant women without thrombosis. Using these inputs, the required sample size was calculated to be 126, with 42 cases and 84 controls.

Data collection procedure

Data were collected through systematic in-person interviews and detailed medical record reviews using a semi-structured questionnaire. The questionnaire was designed to gather comprehensive information on demographic characteristics, anthropometric measurements, medical history, and clinical parameters. Socioeconomic status was assessed using the modified Kuppuswamy scale, which has been validated for use in urban and rural Indian populations [15].

Pre-pregnancy weight and height measurements were used to calculate BMI. Following WHO Asia Pacific guidelines, obesity in Asian populations was defined as BMI ≥25 kg/m^2^. The WHO classification of obesity was also documented, categorizing patients into Class I (BMI: 30-34.9 kg/m^2^), Class II (BMI: 35-39.9 kg/m^2^), and Class III (BMI: ≥40 kg/m^2^) obesity [16].

Patients underwent comprehensive clinical evaluations, including detailed symptom assessment, physical examination, and relevant imaging studies. Associated risk factors, including parity, gestational age, multiple pregnancies, period of immobility, puerperal infection, and previous history of thrombosis, were documented. The diagnosis of different types of thrombosis followed standardized protocols using specific imaging modalities: ultrasound Doppler for DVT, CT angiography for pulmonary thromboembolism, and magnetic resonance venography for CVT.

Laboratory investigations included complete blood count, blood grouping, thrombophilia screening, and antiphospholipid antibodies (APLA) status, with additional tests performed based on clinical presentation and type of thrombosis. Treatment modalities were documented, including the use of unfractionated heparin (UFH), low molecular weight heparin (LMWH), or combination therapy approaches. Patient outcomes were categorized as complete resolution, improvement with ongoing treatment, residual thrombosis/recurrence, or mortality.

Ethical considerations

Prior to conducting the study, we obtained approval from the institutional ethics committee of Government Medical College, Thiruvananthapuram (approval number: 01/10/2017/MCT). A full description was communicated to each person taking part, and their written consent was obtained before data collection began.

Data analysis

We have analyzed the collected data in SPSS Statistics version 26.0 (IBM Corp. Released 2019. IBM SPSS Statistics for Windows, Version 26.0. Armonk, NY: IBM Corp). The mean and standard deviation were used to express continuous variables, while frequencies and percentages were used to present categorical variables. The chi-square test of independence was employed to examine relationships between categorical variables, specifically focusing on comparing the distribution of comorbidities between cases and controls, analyzing associations between obesity and thrombosis types, studying thrombosis distribution across antenatal and postnatal periods, and evaluating treatment outcomes across different groups. In cases where expected cell frequencies fell below five, Fisher's exact test was utilized as an alternative. A p-value of less than 0.05 was considered statistically significant. 

## Results

The mean age of cases was higher (26.9±4.9 years) compared to controls (24.3±3.2 years). Similarly, the mean BMI was higher among cases (24.7±3.6 kg/m^2^) than controls (22.1±2.4 kg/m^2^). Among cases, 23 (54.8%) were nulliparous and 19 (45.2%) were multiparous, while in the control group, 33 (39.3%) were nulliparous and 51 (60.7%) were multiparous. The socio-economic status distribution among cases showed four participants in the upper middle (9.5%), 17 in the lower middle (40.5%), 13 in the upper lower (31.0%), and eight in the lower class (19.0%). Among controls, two participants were in the upper middle (2.4%), 33 in the lower middle (39.3%), 39 in the upper lower (46.4%), and 10 in the lower class (11.9%). Table 1 shows the demographic and clinical characteristics of cases and controls during pregnancy and the postpartum period.

**Table 1:** Demographic and clinical characteristics of study participants with and without thrombosis during pregnancy and postpartum period

Variables		Case (42)		Control (84)	
		n	%	n	%
Age		26.9±4.9		24.3±3.2	
BMI		24.7±3.6		22.1±2.4	
Parity	Nullipara	23	54.8	33	39.3
	Multipara	19	45.2	51	60.7
Socio-economic status	Upper middle	4	9.5	2	2.4
	Lower middle	17	40.5	33	39.3
	Upper lower	13	31	39	46.4
	Lower	8	19	10	11.9

The distribution of BMI classes showed significant differences between cases and controls (p<0.001), as shown in Table 2. Among cases, two (4.8%) were underweight, 15 (35.7%) had a normal BMI, one (2.4%) was overweight, 21 (50.0%) had Class I obesity, and three (7.1%) had Class II obesity. In the control group, two (2.4%) were underweight, 58 (69.0%) had normal BMI, 10 (11.9%) were overweight, 14 (16.7%) had Class I obesity, and none had Class II obesity. Notably, Class I obesity was more prevalent among cases (50.0%) compared to controls (16.7%), and Class II obesity was exclusively found in cases.

**Table 2:** Distribution of study participants according to BMI classification during pregnancy and postpartum period BMI, body mass index

BMI class		Case (42)		Control (84)		Total (126)		Fisher's exact test value	p-value
		N	%	N	%	N	%		
Underweight	≤18.5	2	4.8	2	2.4	4	3.2	25.979	<0.001
Normal	18.6-22.9	15	35.7	58	69	73	57.9		
Overweight	23-24.9	1	2.4	10	11.9	11	8.7		
Class I obesity	25-29.9	21	50	14	16.7	35	27.8		
Class II obesity	≥30	3	7.1	0	0	3	2.4		

Table 3 shows the clinical characteristics and risk factors among cases and controls during pregnancy and the postpartum period. Among cases versus controls, blood group distribution showed A+ in 10 (23.8%) versus 18 (21.4%), B+ in 15 (35.7%) versus 22 (26.2%), AB+ in four (9.5%) versus five (6.0%), O+ in nine (21.4%) versus 17 (20.2%), and Rh negative in four (9.5%) versus 22 (26.2%). Nulliparity was observed in 23 (54.8%) cases and 33 (39.3%) controls. In the antenatal period, significantly more cases occurred at ≤28 weeks gestation, with 19 (70.4%) cases compared to 23 (42.6%) controls. During the postnatal period, thrombosis was notably more frequent between 15 and 42 days, with nine (60.0%) cases compared to two (6.7%) controls. Multiple pregnancies were present in two (4.8%) cases and two (2.4%) controls. Abruption was noted in one (2.4%) case and none of the controls. Assisted reproductive technology (ART) therapy was utilized in two (4.8%) cases and one (1.2%) control. Cases showed higher rates of immobility and puerperal infection, each affecting five patients (11.9%), while no controls experienced these complications. Previous history of thrombosis was documented in one (2.4%) case with no occurrences in controls.

**Table 3:** Clinical characteristics and risk factors among cases and controls during pregnancy and postpartum period *Statistically significant at p-value less than 0.05. ^**^Chi-square test was used. ART, assisted reproductive technology

Variables		Case (42)		Control (84)		Fisher's exact test value	p-value
		n	%	n	%		
Blood group	A+	10	23.8	18	21.4	5.225	0.265
	B+	15	35.7	22	26.2		
	AB+	4	9.5	5	6		
	O+	9	21.4	17	20.2		
	Rh neg	4	9.5	22	26.2		
Parity	Nullipara	23	54.8	33	39.3	2.716^**^	0.099
	Multipara	19	45.2	51	60.7		
Period of gestation	≤28 weeks	19	70.4	23	42.6	5.560^**^	0.01^*^
	>28 weeks	8	29.6	31	57.4		
Postnatal period	15-42 days	9	60	2	6.7	15.400	0.001^*^
	1-14 days	6	40	28	93.3		
Multiple pregnancy	Yes	2	4.8	2	2.4	0.516	0.472
	No	40	95.2	82	97.6		
Abruption	Yes	1	2.4	0	0	2.016	0.156
	No	41	97.6	84	100		
ART therapy	Yes	2	4.8	1	1.2	1.537	0.215
	No	40	95.2	83	98.8		
Period of immobility	Yes	5	11.9	0	0	10.413	0.001^*^
	No	37	88.1	84	100		
Puerperal infection	Yes	5	11.9	0	0	10.413	0.001^*^
	No	37	88.1	84	100		
Previous history of thrombosis	Yes	1	2.4	0	0	2.016	0.156
	No	41	97.6	84	100		

This study analyzed various comorbidities between the case (n=42) and control (n=84) groups, as shown in Table 4. Among the cases, anemia was present in nine (21.4%) participants compared to 13 (15.5%) in the control group. Hyperemesis was equally distributed between both groups, affecting 11 (26.2%) cases and 22 (26.2%) controls. Diabetes mellitus was observed in 13 (31.0%) cases versus 20 (23.8%) controls, while hypertension was present in 11 (26.2%) cases compared to 20 (23.8%) controls. Less common conditions included thrombophilia, present in one (2.4%) case and none in the control group, and connective tissue disease, affecting three (7.1%) cases and three (3.6%) controls. Hyper-homocysteinemia was identified in one (2.4%) case with no occurrences in the control group. Notably, APLA positivity showed a statistically significant difference (p=0.001) between the groups, with five (11.9%) positive cases compared to none in the control group. Statistical analysis using chi-square tests revealed that most comorbidities were not significantly different between the groups (p>0.05), except for APLA status.

**Table 4:** Distribution of comorbidities among cases and controls in pregnancy *p<0.05 was considered statistically significant. **Fisher's exact test was used. APLA, antiphospholipid antibodies; χ2, chi-square test

Variables		Case (42)		Control (84)		χ2 value	p-value
		n	%	n	%		
Anemia	Yes	9	21.4	13	15.5	0.688	0.407
	No	33	78.6	71	84.5		
Hyperemesis	Yes	11	26.2	22	26.2	0.000	1.000
	No	31	73.8	62	73.8		
Diabetes mellitus	Yes	13	31	20	23.8	0.739	0.39
	No	29	69	64	76.2		
Hypertension	Yes	11	26.2	20	23.8	0.086	0.77
	No	31	73.8	64	76.2		
Thrombophilia	Yes	1	2.4	0	0	2.016^**^	0.156
	No	41	97.6	84	100		
Connective tissue disease	Yes	3	7.1	3	3.6	0.788^**^	0.375
	No	39	92.9	81	96.4		
Hyper-homocystinemia	Yes	1	2.4	0	0	2.016^**^	0.156
	No	41	97.6	84	100		
APLA	Positive	5	11.9	0	0	10.413^**^	0.001^*^
	Negative	37	88.1	84	100		

Analysis of the presenting symptoms among the study participants revealed a distinct pattern of clinical manifestations (Table 5). The most predominant symptom cluster was leg pain accompanied by redness and edema, affecting 29 (69.0%) patients. This was followed by neurological symptoms, including blurring of vision, headache, and diplopia, which were reported in eight (19.0%) patients. Neurological and systemic manifestations in the form of seizures, vomiting, and fever were observed in four (9.5%) patients. Cardiopulmonary symptoms, specifically chest pain and dyspnea, were the least common, present in only one (2.4%) patient.

**Table 5:** Distribution of presenting symptoms among cases (n=42)

Most common symptom	Frequency	Percentage (%)
Leg pain, redness, edema	29	69
Blurring, headache, diplopia	8	19
Chest pain, dyspnea	1	2.4
Seizure, vomiting, fever	4	9.5
Total	42	100

Table 6 shows the distribution of thrombotic events by anatomical location. DVT was the most frequent type, occurring in 29 (69.0%) patients, representing more than two-thirds of all cases. CVT was the second most common manifestation, affecting 12 (28.6%) patients. PTE was the least common, documented in only one (2.4%) patient.

**Table 6:** Distribution of thrombotic events by anatomical location DVT, deep vein thrombosis; CVT, cortical venous thrombosis; PTE, pulmonary thromboembolism

Type of thrombosis	Frequency	Percentage (%)
DVT	29	69
CVT	12	28.6
PTE	1	2.4
Total	42	100

Table 7 shows the anatomical distribution of thrombosis sites in cases. The ileo-femoral region was the most affected site, accounting for 26 (61.9%) cases, representing two-thirds of all thrombotic events. The second most frequent location was the sigmoid and sagittal sinuses, involving seven (16.7%) patients, followed by popliteal and short femoral vessels, which were affected in five (11.9%) cases. Tibial vessel thrombosis was observed in two (4.8%) patients. The least common sites were the transverse sinus and pulmonary vessels, each affecting one (2.4%) patient. This distribution demonstrates a clear predominance of lower extremity thrombosis, particularly in the ileo-femoral region.

**Table 7:** Anatomical distribution of thrombosis sites in cases (n=42)

Site of thrombosis	Frequency	Percentage (%)
Ileo-femoral	26	61.9
Popliteal and short femoral	5	11.9
Tibial	2	4.8
Sigmoid and sagittal	7	16.7
Transverse	1	2.4
Pulmonary vessels	1	2.4
Total	42	100

The analysis of thrombosis types in relation to obesity status revealed notable patterns, as shown in Table 8. Among patients with DVT, 17 (58.6%) were obese compared with 12 (41.4%) non-obese patients, showing a statistically significant association (p=0.001). CVT showed an equal distribution between obese and non-obese groups, with six (50.0%) patients in each category, and this association was not statistically significant. A single case of pulmonary embolism was observed in an obese patient (100%), though this finding was not statistically significant. These results suggest a particularly strong association between obesity and DVT.

**Table 8:** Association between obesity status and different types of thrombosis among study participants ^*^p<0.05 was considered statistically significant. DVT, deep vein thrombosis; CVT, cortical venous thrombosis; PTE, pulmonary thromboembolism

Type of thrombosis	Obese		Non-obese		Total		χ2 value	p-value
	n	%	n	%	n	%		
DVT	17	58.6	12	41.4	29	100	14.488	0.001^*^
CVT	6	50	6	50	12	100	2.479	0.115
PTE	1	100	0	0	1	100	2.334	0.127

Table 9 shows the distribution of thrombotic events between antenatal and postnatal periods among pregnant women. DVT showed a predominant occurrence during the antenatal period, with 21 (72.4%) cases compared to eight (27.6%) cases in the postnatal period, though this difference was not statistically significant. CVT demonstrated an opposite trend, with a higher frequency in the postnatal period affecting seven (58.3%) patients compared to five (41.7%) patients in the antenatal period, but this difference also did not reach statistical significance. A single case (100%) of pulmonary embolism occurred during the antenatal period. These findings suggest that, while DVT tends to occur more frequently during the antenatal period, CVT shows a slight predilection for the postnatal period, although none of these associations achieved statistical significance in our study population.

**Table 9:** Distribution of thrombotic events between antenatal and postnatal periods among pregnant women DVT, deep vein thrombosis; CVT, cortical venous thrombosis; PTE, pulmonary thromboembolism

Type of thrombosis	Antenatal period		Postnatal period		Total		χ2 value	p-value
	n	%	n	%	n	%		
DVT	21	72.4	8	27.6	29	100	1.084	0.298
CVT	5	41.7	7	58.3	12	100	2.956	0.086
PTE	1	100	0	0	1	100	0.56	0.454

The analysis of anticoagulation treatment protocols revealed varied approaches to managing thrombosis among cases (Table 10). UFH was the most frequent treatment modality, administered to 19 (45.2%) patients, representing nearly half of the study population. Combined therapy using both LMWH and UFH was employed in 12 (28.6%) cases, while LMWH monotherapy was used in 11 (26.2%) patients. This distribution indicates that UFH, either alone or in combination, was the predominant treatment choice, being used in approximately three-quarters of all cases (73.8%). The selection of treatment protocols reflected individual patient characteristics, severity of thrombosis, and specific clinical scenarios encountered during management.

**Table 10:** Distribution of anticoagulation treatment protocols among study cases (N=42) LMWH, low molecular weight heparin; UFH, unfractionated heparin

Treatment protocol used for cases	Frequency	Percentage
LMWH	11	26.2
UFH	19	45.2
Combined (LMWH +UFH)	12	28.6
Total	42	100

Table 11 shows the distribution of clinical outcomes among thrombosis patients following anticoagulation therapy. The majority of patients showed favorable outcomes, with 26 (61.9%) cases showing ongoing improvement in continued treatment at the time of assessment. Complete resolution was achieved in 14 (33.3%) patients, indicating successful treatment outcomes in these cases. Adverse outcomes were relatively rare, with residual thrombosis or recurrence observed in one (2.4%) patient. Similarly, mortality was documented in one (2.4%) case. Overall, the outcomes were positive, with 40 (95.2%) patients either achieving complete resolution or showing improvement with ongoing treatment, while adverse outcomes occurred in only two (4.8%) cases.

**Table 11:** Clinical outcomes of cases of thrombosis following treatment (N=42)

Outcome	Frequency	Percentage
Resolved completely	14	33.3
Resolving by ongoing treatment	26	61.9
Residual thrombosis/recurrence	1	2.4
Mortality (death)	1	2.4
Total	42	100

## Discussion

This study provides critical insights into the association between obesity and thrombosis during pregnancy and the postpartum period in a tertiary care center in Kerala, India. Our findings demonstrate a significant relationship between elevated BMI and thrombotic events, with particular emphasis on DVT during pregnancy and the postpartum period.

The demographic analysis revealed that cases had a higher mean BMI (24.7±3.6 kg/m^2^) compared with controls (22.1±2.4 kg/m^2^), highlighting the role of increased body mass in thrombotic risk. In the case group, 21 patients (50.0%) presented with Class I obesity, and three patients (7.1%) with Class II obesity. The control group included 14 patients (16.7%) with Class I obesity and no patients (0%) with Class II obesity, revealing a significant difference in BMI class distribution between groups. These findings align with the few studies that have established obesity as an independent risk factor for thrombotic events during pregnancy. Mahmoud et al. demonstrated that pre-pregnancy overweight and obesity significantly increase the long-term risk of VTE, with risk increasing proportionally with BMI [6]. Similarly, Hotoleanu's comprehensive review revealed that obesity creates a prothrombotic state through multiple mechanisms [3].

Our study found a striking prevalence of Class I obesity among cases (21, 50.0%) compared to controls (14, 16.7%), with Class II obesity exclusively present in cases. This distribution pattern strongly supports the findings of Wu et al., who demonstrated a significant association between maternal weight and VTE risk [2]. The mechanisms underlying this association are complex and multifactorial. As explained by Yang et al., obesity creates a prothrombotic state through various pathways, including increased inflammation, oxidative stress, and alterations in adipokine profiles [7]. Additionally, recent research by Manna Li et al. has shown that adipose tissue in obese individuals secretes inflammatory cytokines that promote endothelial dysfunction and enhance platelet activation, further contributing to thrombotic risk [17].

Our study demonstrated a strong correlation between obesity and DVT, with 17 DVT cases (58.6%) occurring in obese patients. This observation is consistent with two key studies: James et al. demonstrated that obesity independently increased the risk of pregnancy-related VTE four-fold in obese women compared to non-obese women, while Jacobsen et al. found that a pre-pregnancy BMI ≥25 kg/m^2^ significantly elevated the risk of pregnancy-related VTE [18,19]. These findings align with Malinowski et al.'s work, which explained that obesity enhances DVT risk during pregnancy through multiple mechanisms, particularly venous stasis and hypercoagulability [8].

The timing of thrombotic events showed distinct patterns, with 19 antenatal cases (70.4%) occurring at ≤28 weeks gestation. This finding differs from Jacobsen et al.'s work, which found a more even distribution throughout pregnancy [20]. However, our observation of increased thrombotic events between 15 and 42 days postpartum aligns with James et al.'s identification of the postpartum period as a time of heightened thrombotic risk [21]. The ACOG practice bulletin explains that pregnancy itself is a hypercoagulable state with increases in fibrinogen and coagulation factors, decreased protein S levels, and decreased fibrinolysis [22]. These physiological changes begin early in pregnancy, and when combined with obesity (which independently increases thrombotic risk through inflammatory mediators and altered coagulation factors) can significantly increase the risk of thromboembolism from early gestation [22].

The analysis of risk factors revealed several significant associations. The presence of immobility (5 cases, 11.9%) and puerperal infection (5 cases, 11.9%) aligns with Anderson and Spencer's comprehensive review [23]. The significant association of APLA positivity with thrombotic events (5 cases vs. 0 controls) supports findings by Greer regarding the importance of thrombophilia screening in high-risk pregnancies [24].

Our study found that 54.8% of thrombotic events occurred in nulliparous women (23 cases), a finding that contrasts with established patterns in the literature. In their United Kingdom population-based cohort study, Sultan et al. demonstrated that women with two or more previous pregnancies had a significantly higher risk of VTE compared with nulliparous women [25]. This difference between our findings and the UK study suggests that the relationship between parity and thrombotic risk may be influenced by population-specific or regional factors.

The anatomical distribution of thrombotic events in our study showed that DVT was the predominant manifestation (29 cases, 69.0%), particularly affecting the ileo-femoral region (26 cases, 61.9%). This pattern is consistent with the findings of Devis and Knuttinen, who noted that pregnancy-associated DVT commonly affects the left lower extremity, particularly the iliofemoral region [26]. The predilection for left-sided thrombosis is attributed to the anatomical compression of the left iliac vein by the right iliac artery, combined with the compression from the enlarging uterus [27].

The relationship between obesity and specific types of thrombosis revealed that 17 DVT cases (58.6%) occurred in obese patients, showing a statistically significant association. This finding supports the work of Malinowski et al., who emphasized the need for specific attention to VTE risk in obese pregnant women [8]. The equal distribution of CVT between obese and non-obese groups suggests that obesity might play a different role in cerebral venous thrombosis compared to PVT.

Treatment outcomes in our study were favorable, with 40 patients (95.2%) achieving either complete resolution or showing improvement with ongoing treatment. This positive outcome rate supports CheYaakob et al.'s findings regarding the effectiveness of anticoagulation therapy in pregnancy-related DVT [28]. UFH was the most frequent treatment modality, administered to 19 (45.2%) patients, representing nearly half of the study population. Combined therapy using both LMWH and UFH was employed in 12 (28.6%) cases. Our treatment protocol aligns with current guidelines for managing pregnancy-associated thrombosis in obese patients [29].

The socioeconomic distribution of cases in our study, with a predominance in the lower middle- and upper-lower classes, suggests potential healthcare access and awareness disparities. This observation is particularly relevant in the context of Kerala's maternal health landscape, where thrombosis has been identified as a significant contributor to maternal deaths, as reported in the CRMD [11].

These findings have important implications for clinical practice, particularly in identifying high-risk patients and implementing appropriate preventive measures. As emphasized by Navti and Pavord, the management of VTE in pregnant obese individuals requires careful consideration of multiple risk factors and appropriate prophylaxis strategies [9]. The strong association between obesity and thrombotic events, particularly DVT, underscores the importance of weight management before and during pregnancy, as well as careful monitoring during both antenatal and postpartum periods.

Limitations

This study has fewer limitations that should be considered when interpreting the results. Being a single-center study conducted at a tertiary care center in Kerala, the findings may not be generalizable to other populations or healthcare settings. The small sample size of 42 cases and 84 controls may have limited the statistical power to detect associations with less common risk factors and could have affected the robustness of the statistical analyses. The study lacks information about the duration of follow-up, which is crucial for assessing long-term outcomes of thrombotic events and treatment efficacy. Additionally, important confounding variables, such as lifestyle factors, dietary habits, and physical activity levels, which could influence both obesity and thrombosis risk, were not addressed. The absence of multivariate analyses to adjust for potential confounding factors further limits the interpretation of the associations observed between risk factors and thrombotic events.

Recommendations

Future research should focus on conducting larger, multi-center studies with longer follow-up periods to enhance the generalizability and strength of findings. Weight management programs and pre-pregnancy counseling services should be integrated into standard antenatal care, particularly focusing on obesity as a modifiable risk factor. Early mobilization strategies and strict infection control protocols during the puerperal period should be established to minimize thrombotic risk. Additionally, developing standardized thromboprophylaxis guidelines specifically for obese pregnant women could help prevent adverse outcomes. Regular monitoring and early intervention protocols for high-risk patients should be institutionalized to ensure timely detection and management of potential thrombotic events.

## Conclusions

This study demonstrates that obesity is a significant risk factor for thrombotic events during pregnancy and the postpartum period, particularly for deep vein thrombosis. The high prevalence of Class I and II obesity among cases, coupled with the strong association between obesity and DVT, underscores the importance of weight management in preventing pregnancy-related thrombosis. Additional risk factors, including immobility, puerperal infection, and APLA positivity, were also identified as significant contributors. While DVT predominantly occurred during the antenatal period, especially in the ileo-femoral region, CVT showed a slight predilection for the postpartum period. The favorable treatment outcomes observed suggest that early recognition and appropriate anticoagulation therapy can effectively manage pregnancy-related thrombosis, though preventive strategies focusing on modifiable risk factors should be prioritized.
